# Camptothecin Encapsulated in β-Cyclodextrin-EDTA-Fe_3_O_4_ Nanoparticles Induce Metabolic Reprogramming Repair in HT29 Cancer Cells through Epigenetic Modulation: A Bioinformatics Approach

**DOI:** 10.3390/nano11123163

**Published:** 2021-11-23

**Authors:** Aisha Farhana, Avin Ee-Hwan Koh, Pooi Ling Mok, Abdullah Alsrhani, Yusuf Saleem Khan, Suresh Kumar Subbiah

**Affiliations:** 1 Department of Clinical Laboratory Sciences, College of Applied Medical Sciences, Jouf University, Sakaka 72388, Saudi Arabia; rachelmok2005@gmail.com (P.L.M.); afalserhani@ju.edu.sa (A.A.); 2 Department of Biomedical Science, Faculty of Medicine and Health Sciences, Universiti Putra Malaysia (UPM), Serdang 43400, Malaysia; avin.keh@gmail.com; 3 Department of Anatomy, College of Medicine, Jouf University, Sakaka 72388, Saudi Arabia; dryusufkhan@gmail.com; 4 Department of Medical Microbiology and Parasitology, Universiti Putra Malaysia (UPM), Serdang 43400, Malaysia; 5 Centre for Materials Engineering and Regenerative Medicine, Bharath Institute of Higher Education and Research, Bharath University, Selaiyur, Chennai 600073, India; 6 Institute of Bioscience, Universiti Putra Malaysia (UPM), Serdang 43400, Malaysia

**Keywords:** metabolic reprogramming, epigenetic modulation, colon cancer, nanoparticles, transcriptome analysis

## Abstract

Cancer progresses through a distinctive reprogramming of metabolic pathways directed by genetic and epigenetic modifications. The hardwired changes induced by genetic mutations are resilient, while epigenetic modifications are softwired and more vulnerable to therapeutic intervention. Colon cancer is no different. This gives us the need to explore the mechanism as an attractive therapeutic target to combat colon cancer cells. We have previously established the enhanced therapeutic efficacy of a newly formulated camptothecin encapsulated in β-cyclodextrin-EDTA-Fe_3_O_4_ nanoparticles (CPT-CEF) in colon cancer cells. We furthered this study by carrying out RNA sequencing (RNA-seq) to underscore specific regulatory signatures in the CPT-CEF treated versus untreated HT29 cells. In the study, we identified 95 upregulated and 146 downregulated genes spanning cellular components and molecular and metabolic functions. We carried out extensive bioinformatics analysis to harness genes potentially involved in epigenetic modulation as either the cause or effect of metabolic rewiring exerted by CPT-CEF. Significant downregulation of 13 genes involved in the epigenetic modulation and 40 genes from core metabolism was identified. Three genes, namely, *DNMT-1*, *POLE3*, and *PKM-2*, were identified as the regulatory overlap between epigenetic drivers and metabolic reprogramming in HT29 cells. Based on our results, we propose a possible mechanism that intercepts the two functional axes, namely epigenetic control, and metabolic modulation via CPT-CEF in colon cancer cells, which could skew cancer-induced metabolic deregulation towards metabolic repair. Thus, the study provides avenues for further validation of transcriptomic changes affected by these deregulated genes at epigenetic level, and ultimately may be harnessed as targets for regenerating normal metabolism in colon cancer with better treatment potential, thereby providing new avenues for colon cancer therapy.

## 1. Introduction

Metabolic reprogramming is established as a hallmark of cancer progression. The initial understanding of cancer as a metabolic disease was substantiated by the demonstration of glycolytic pathway abnormalities in cancer, described as the Warburg effect. Cancer proliferates through selective enhancement or skewing of metabolic activity, which provides a high proliferative index that abates oxidizing environments and cell death mechanisms such as apoptosis [1]. The energy required for enhanced proliferation is met by reprogramming the nutrient acquisition mechanism and metabolic pathways [2]. Most metabolic pathways are observed to be reprogrammed through oncogenic signaling and transcriptional networks in a cell-autonomous control [3]. Both genomic and epigenomic processes are identified to dictate metabolic switching in various cancers [4]. However, there is a synergistic overlap between epigenetic and genetic pathways that propel neoplastic transformations [5]. Most metabolic alterations are shown to be regulated through epigenetic modulations, which exert a substantial effect on gene expression patterns. The metabolites produced through various biochemical pathways are intrinsic substrates and cofactors for enzymes that function in epigenetic modulation and genomic transcription [6]. Hence, it is imperative to conjecture that epigenetic changes, metabolic reprogramming, and transcriptional regulations converge to generate cancerous transformation.

Genetic alterations leading to the development of cancer phenotypes are incorporated through base changes and are usually irreversible. The cancerous proliferation of the cells via genomic mutations generates resilient phenotypes. These hardwired abnormalities incorporated into the genome are daunting for developing effective treatment regimes. On the other hand, the cancer metabolic landscaping mechanisms regulated through epigenetic switches hold more potential to be effectively harnessed as treatment targets [7]. Furthermore, sophisticated techniques have assisted us in understanding cancer as a biologically heterogeneous entity, even within a single subtype. This points toward an interplay of various genetic and epigenetic events mobilizing tumorigenic transformation towards specific cancer types and subtypes [8].

Epigenetic changes influence and are influenced by both intrinsic microenvironments and extrinsic factors in a reversible manner. Cellular growth advantages induced through epigenetic events may develop a state of enhanced proliferation, usually selected for in a tissue. This characteristic may augment progressive uncontrolled growth as observed in cancer. The metabolic requirement of proliferating cancer cells is met through rewiring of the normal metabolism towards fulfilling the needs of cell growth [9]. Hence, the intrinsic cellular microenvironment adjusted to meet the metabolic needs of enhanced cellular growth potentially alters chromatin structure by selectively regulating the metabolite supply towards chromatin modifier enzymes [10]. Hence, a chromatin-metabolic coupling mechanism arbitrates the overall reprogramming of the cell towards a specific phenotype. This phenomenon plausibly functions through epigenetic modulations that integrate the two mechanisms. This epigenetic plasticity of the cells caters to the metabolic need to maintain a high proliferative index through altering chromatin structure [11].

Epigenetic modifications affect miRNA expression, DNA methylation, and histone acetylation status to silent genes involved in tumor suppressors, DNA repair, modulation of cellular metabolic and regulatory pathways. The functional silencing of these genes would lead to cancer initiation and progression [12,13]. Epigenetic silencing and altered metabolic regulation exert their effect on the generation of oncometabolites that sustain cancer progression [14]. Metabolic accumulation of short-chain fatty acids is shown to induce morphological changes in cells, cell cycle arrest, and apoptosis [15]. Similarly, metabolic channeling of aerobic glycolysis and oxidative phosphorylation intermediates such as glucose and glutamine towards the generation of oncometabolites has been reported [16].

As a therapeutic mechanism, selective induction of suppressor genes through chemical modulation has proven to be successful in cell culture and animal models. Some of these chemical modulators have also shown success in clinical trials, one of them being camptothecin (CPT). CPT is a selective topoisomerase I inhibitor proposed to function through inhibition of NO biosynthesis [17,18]. It is a major anticancer drug that is effective for many cancers, including ovarian and colorectal cancers. Our laboratory has successfully established that magnetic nanocarrier conjugated CPT, CPT conjugated with β-cyclodextrin and iron NPs (Fe_3_O_4_), and cross-linked using EDTA (CPT-CEF), has a higher efficacy as an anticancer drug as compared to CPT alone. The enhanced potential of CPT-CEF as a drug is subjected to its increased solubility and selective targeting. CPT-CEF induces the activation of the apoptotic pathway and finally cell death [19,20]. Apart from CPT, β-cyclodextrins have been used in the encapsulation of several other drugs or compounds. Some of the most recent examples include antibacterial electrospun nanofibers [21], synthetic N-acyl-l-homoserine lactones for quorum sensing inhibition [22], and even hydrogel-based microneedles for water-insoluble drug delivery [23].

In the present study, we carried out RNA sequencing (RNA-seq) to gain insights into the pathways that are selectively regulated by CPT-CEF. We focused our study on identifying expression patterns that showed epigenetic modulations and hence could be used as softwired cancer therapeutic targets. RNA-seq followed by bioinformatics analyses identified genes strategic to metabolic switching, which is a vital nexus towards tumorigenic transformations. The cellular alterations that levitate a normal phenotype towards cancerous transformation functioning via metabolic reprogramming essentially work through epigenetically controlled bypass switches and serve as vulnerable targets for promising therapeutic interventions.

## 2. Results

### 2.1. Visualization of the CPT-CEF-Treated HT29 Colon Cancer Cells through Over-Representation Analysis from RNA Sequencing Data

**Figure 1:**
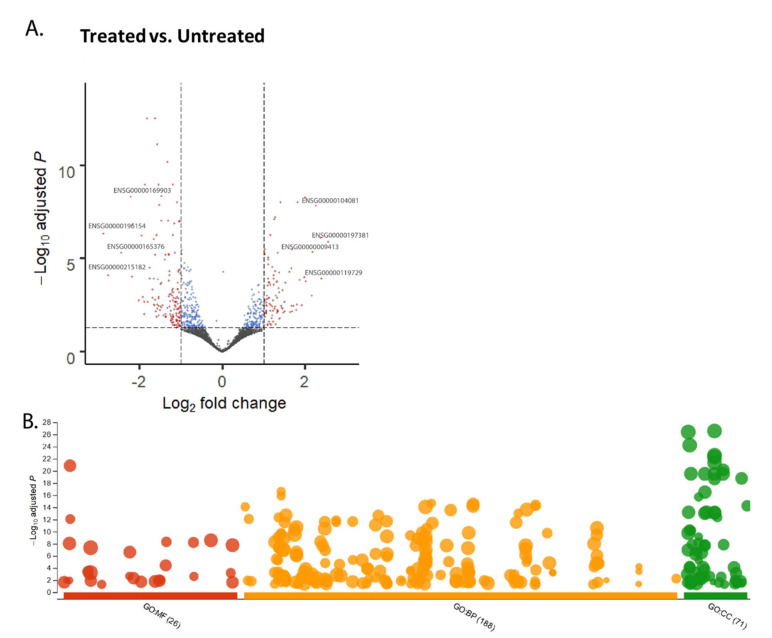
Over-representation analysis and visualization of genes in CPT-CEF-treated colon cancer cells. (**A**) A total of 95 upregulated and 146 downregulated genes were obtained from the RNA-seq data via DESeq2, as shown by the volcano plot (adj *p* < 0.05, FC > 2.0). (**B**) Over-representation analysis was performed using g:Profiler and the data were categorized based on gene ontology (GO) subontologies and plotted as a scatterplot of hits against −log10 adj *p*-value. The analysis yielded 26 GO terms based on molecular functions (MF), 188 GO terms based on biological processes (BP), and 71 GO terms based on cellular components (CC). The scatterplot was plotted as against the databases.

The RNA sequencing data obtained from CPT-CEF treated and untreated HT29 cells was processed through DESeq2 and is shown by the volcano plot (adj *p* < 0.05, FC > 2.0). This resulted in the identification of 95 upregulated and 146 downregulated genes (Figure 1A). The over-representation analysis carried out using g:Profiler and data thus obtained categorized based on gene ontology (GO) subontologies yielded 26 GO terms that were based on molecular functions (MF), 188 GO terms based on biological processes (BP), and 71 GO terms based on cellular components (CC). The can be observed in the scatterplot provided in Figure 1B. Discrete top 10 GO subontologies terms were tabulated in Table 1. Most of these overrepresented terms in MF subontology pointed towards the downregulation of genes that constitute the synthetic function within the cells, such as the structural constituent of the ribosome, rRNA binding, cell adhesion molecule binding, molecular complex binding, macromolecular complex binding, etc. (listed in Table 1). CC subontology terms point towards genes involved in the mitochondrial envelope, focal adhesion, membrane-enclosed lumen, and ribonucleoprotein complex. The terms constituting the biological function indicated downregulated genes such as SRP-dependent co-translational protein targeting to membrane, metabolic processes, nuclear-transcribed mRNA catabolic process, nonsense-mediated decay, cellular component organization, or biogenesis, etc.

### 2.2. Identification of Genes Involved in Epigenetic Modification Differentially Regulated upon CPT-CEF Treatment of HT29 Colon Cancer Cells

The filtered data (adj *p* < 0.05, FC > 2.0) obtained from over-representation analysis was used for data mining genes that are involved in epigenetic modification, using EpiFactors as a reference database for analysis. A list of 13 genes (Table 2) from the filtered dataset are involved in epigenetic modifications and are differentially induced in CPT-CEF treated cells, as indicated by fold change (Table 2). Most of the genes differentially regulated in CPT-CEF treated cells belong to chromatin remodeling and histidine modification pathways. Among the upregulated genes were up CHD6, ARID48 and KD5A. Some significant genes downregulated upon treatment with CPT-CEF were HMGB1, MTA1, HDGF, and DNMT1.

### 2.3. Identification of Enriched Pathways in CPT-CEF Treated HT29 Cancer Cells

**Figure 2:**
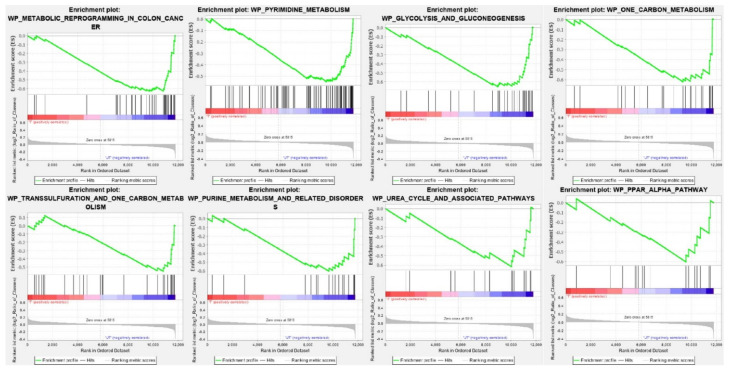
Gene set enrichment analysis (GSEA) of CPT-CEF-treated colon cancer cells. The GSEA tool was used to produce enrichment plots of statistically significant gene sets/pathways. Top gene sets involved in cancer metabolic reprogramming were presented with negative enrichment scores. In other words, the selected pathways were underexpressed in CPT-CEF-treated colon cancer cells, and hence showed a higher correlation with the untreated cells (adj *p* < 0.05).

**Figure 3:**
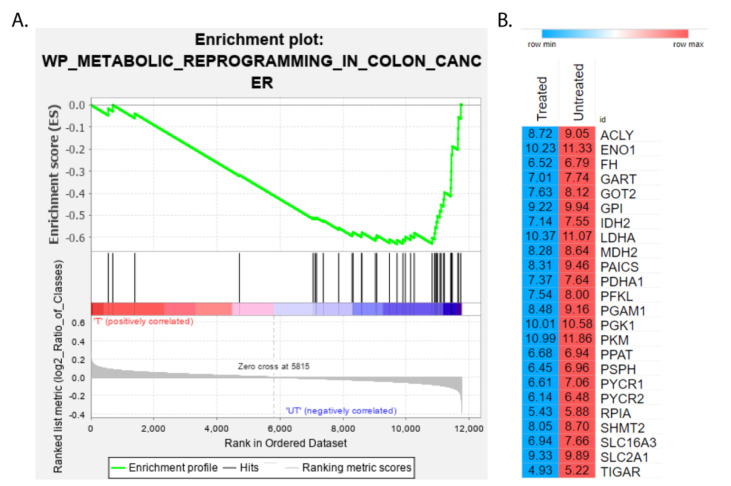
A GSEA enrichment plot of the colon cancer metabolic reprogramming pathway along with a heatmap of the genes that contribute to the plot’s leading edge in CPT-CEF-treated colon cancer cells. (**A**). The GSEA tool was used to produce an enrichment plot of the colon cancer metabolic reprogramming pathway (normalized enrichment score = −2.28) of which, the negative enrichment score (−0.51935) indicated a negative relation (adj *p* < 0.05). (**B**). The leading edge comprised of 24 genes that contributed the highest running scores for this pathway, as shown by the heatmap. The mean normalized count data for each gene between CPF-CEF-treated and untreated samples were plotted. Red: overexpression; blue: downregulation.

For the identification of pathways that are selectively enriched in the CPT-CEF treated HT29 cancer cells, GSEA analysis was performed using the WikiPathways database as a reference (adj *p* < 0.05). A list of the top 20 enriched pathways in CPT-CEF-treated colon cancer cells was obtained upon analysis. The pathways are tabulated in Table 3. Pathways identified from the analysis played a role in metabolic reprogramming, glycolysis, and gluconeogenesis, purine and pyrimidine metabolism, etc. Enrichment plots for the most significant pathways were obtained using the GSEA tool and are shown in Figure 2. The GSEA enrichment plot for the colon cancer metabolic reprogramming pathway, with a normalized enrichment score of −2.28 and the negative enrichment score −0.51935, identified 24 genes contributing to the leading edge (Figure 3A). The repertoire of overexpressed and downregulated genes in CPT-CEF treated and untreated HT29 cells is shown by the heat map (Figure 3B). The GSEA-enriched genes involved in the metabolic reprogramming pathway in CPT-CEF treated cancer cells are listed in Table 4. The leading edge, which constitutes 24 genes, includes pyruvate dehydrogenase E1 subunit alpha 1, ATP citrate lyase, fumarate hydratase, malate dehydrogenase 2, lactate dehydrogenase, pyruvate kinase M1/2, etc.

**Figure 4:**
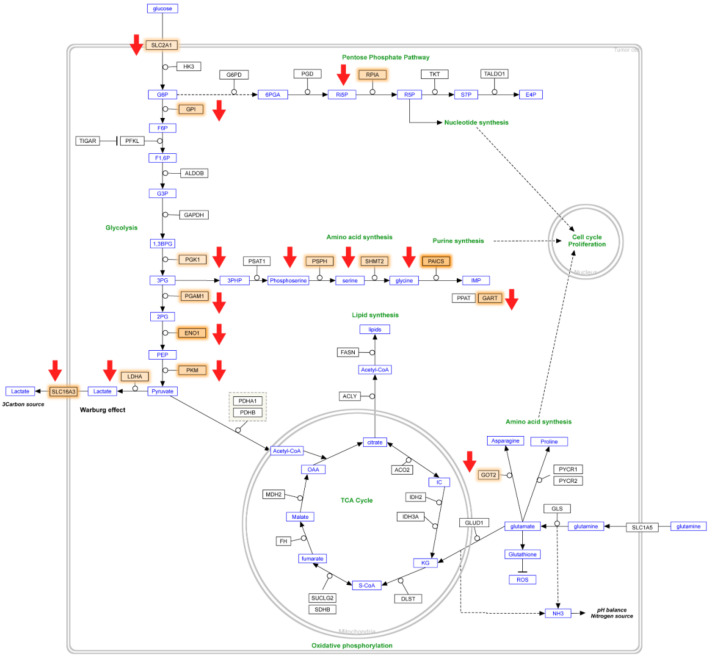
Visualization of significant genes from CPT-CEF-treated colon cancer cells in the cancer metabolic reprogramming pathway. The pathway was extracted from the WikiPathways database, whereas significant genes obtained from the dataset were highlighted in orange. Red arrows indicate downregulation through the action of CPT-CEF.

The GSEA enriched genes significantly contributing to metabolic reprogramming pathway in CPT-CEF-treated colon cancer cells were visualized by extracting the pathway from the WikiPathways database. The genes highlighted in orange (Figure 4) are significantly modulated, which contributes to the downregulation of the metabolic pathway (highlighted via red arrows), as an effect of CPT-CEF treatment. The major pathways that are downregulated concomitantly to the effect of CPT-CEF as compared to the untreated cancerous cells were: (i) glycolytic pathway, with many genes such as glucose transporter (*SLC2A1*), lactate dehydrogenase (*LDHA*), Glucose 6 phosphate isomerase (*GPI*), enolase 1(*ENO1*) and pyruvate kinase (*PKM*) significantly regulated; (ii) Pentose phosphate pathway, with *RPIA* gene, significantly modulated that further affects nucleotide synthesis pathway; (iii) Tricarboxylic acid pathway (TCA) in which the genes isocitrate dehydrogenase (*IDH2*) and *MDH2* (malate dehydrogenase) were significantly enriched; (iv) Amino acid synthesis genes glutamic oxaloacetic transaminase 2 (*GOT2*), pyrolline -5-carboxylase reductase (*PYCR1* and *2*) that function at the final steps to the synthesis of asparagine and proline were enriched in the pathway; (v) Lipid synthetic pathway genes, namely ATP citrate lyase (*ACLY*) and fatty acid synthase (*FASN*), were the enriched genes; (vi) phosphoribosylaminoimidazole carboxylase and phosphoribosylaminoimidazolesuccinocarboxamide synthase (*PAICS*) genes from the purine biosynthesis pathway were significantly enriched (Figure 4).

### 2.4. Identification of Genes with Functional Overlap between Colon Cancer Metabolism and Epigenetic Regulation

The RNA sequencing data were subjected to data mining using EpiFactor as a reference database. Several genes were identified as being involved in cancer metabolism as well as epigenetic modifications (Table 5). Some of the prominent genes were PKM, DNMT1, and POLE3. Besides having a significant role in metabolic pathways, PKM is shown to be involved in histone modification. DNMT is involved in DNA modification and regulation of metabolic genes, and POLE3 acts as a histone chaperone.

## 3. Discussion

The proliferative potential of cancer is propelled and maintained by metabolic rewiring responses directed towards generating energy and metabolites for the sustenance of indefinitely proliferating cells, prone to harsh microenvironments. Tumor cell bioenergetics involve preferential skewing of glycolysis, amino acid, and lipid metabolism associated with increased mitochondrial biogenesis, channelizing substrates into pentose phosphate pathways (PPP), and the synthesis of tumor facilitator biomolecules [24].

Metabolic rewiring in cancers is induced through altered tissue microenvironments, which include increased peroxides, and decreased cellular O_2_ and H^+^ ions resulting from ensuing immune response. Recent evidence points to these changes being sensed through epigenetic mechanisms that regulate metabolic remodeling to generate anabolic precursors that support high proliferation rates [2]. Nonetheless, there exists an integrated cause and effect relationship between epigenetic and metabolic reprogramming [9]. Understanding this link between epigenetic modifications and metabolism reprogramming could plausibly uncover novel molecular targets.

We have previously demonstrated that CPT-CEF has a better efficacy as a cancer drug due to its enhanced solubility and stability in cancer microenvironments, as tested in cancerous HT29 and A549 cells [20]. This formulation is shown to induce growth arrest, subsequently leading to apoptosis when a dose of IC_50_ (133.5 μg/mL) was administered for 48 h [20]. Further, we carried out RNA-seq analysis of CPT-CEF treated versus untreated samples to probe into the genes and pathways that are distinctly modulated by CPT-CEF. Analysis of the data obtained from RNA seq resulted in the identification of genes involved in metabolic reprogramming as one of the top enriched pathways (Table 3, Figure 2). Since metabolic reprogramming is a distinct feature of cancer growth, it is imperative that the metabolic genes that are downregulated in CPT-CEF treated cancer cells are functioning towards stalling the progression of these cells toward further proliferation (Figure 4). Treatment of HT29 cells with CPT-CEF affects genes in the glycolytic pathway, namely glucose importer *SLC2A1* and regulatory enzymes such as *GPI*, *PFKL*, *GAPDH*, *ENO1*, and *PKM2* (Figure 4, Table 4). The downregulation of these genes may have an overall repressive effect on glycolysis, which is utilized by cancer cells towards the generation of metabolites that support tumor progression. Furthermore, genes of the anaerobic glycolysis, i.e., lactate dehydrogenase (*LDH*) and lactate transporter *SLC16A3*, and genes belonging to the pentose phosphate pathway (PPP), namely *RPIA*, were also enriched upon CPT-CEF treatment. We conjecture that repression of these genes may also pose a limiting effect on the movement of metabolic flux. We thus hypothesize that CPT-CEF is effective in repressing glycolysis, a pathway explicitly utilized in metabolic reprogramming of cancer cells. Cancer cells divert glucose flux from aerobic glycolysis to generate energy, wherein glucose is converted to lactate even in the presence of oxygen to sustain cellular growth. Our data indicated an enrichment of pyruvate kinase M (*PKM*) in the CPT-CEF treated cells. *PKM* is highly upregulated during tumorigenesis and functions as a metabolic regulator of the glycolytic pathway in many cancer cells, channelizing glycolytic intermediates away from the respiratory chain towards anaerobic glycolysis [25,26]. Specific downregulation of *PKM* and other genes mentioned above through CPT-CEF may be a metabolic repair mechanism to arrest tumorigenic progression.

Many genes involved in glycolysis, glutaminolysis, and proline metabolism are regulated by the MYC transcription factor [27,28]. We observed that most of the genes that are selectively enriched upon CPT-CEF treatment are directly under the MYC control. MYC specifically induces the genes that direct the glycolytic intermediate towards PPP, facilitating the generation of high concentrations of nicotinamide adenine dinucleotide phosphate (NADPH) and biosynthesis of oncometabolites through the induction of PKM2 over the normally present isoform PKM1 [28,29]. In our study, we observe an altered expression of many glycolytic pathway genes and some amino acid and purine metabolic pathway genes. Besides, the genes of the proline synthesis pathway are also enriched in CPT-CEF treated cells. Proline biosynthetic genes have been previously shown to be regulated by myc [30,31]. Many studies confer that myc induces an alternate expression programming through epigenetic mechanisms, rather than through the recruitment of chromatin regulatory elements [32,33,34]. Together, we hypothesize that CPT-CEF may exert its anticancer effects through the repression vis-à-vis modulation of the *myc* gene, altering the expression of the metabolic genes through an epigenetic mechanism.

The epigenetic and genomic programs of a cell function in an interactive way, mostly through the action of DNA methyltransferases (*DNMT*s), which regulates the methylation status and hence the expression of many genes [35]. In our study, we observe that CPT-CEF treatment downregulates *DNMT1*, one of the enzymes involved in epigenetic modulation (Table 5). DNMT requires S-adenosyl methionine (SAM) as an essential co-substrate for its activity and selectively reinforces transcriptional network that supports epigenetic reprogramming. Colon cancers are sustained by global DNA hypomethylation, plausibly attained through the diminished activity of DNMT1 enzyme [36,37]. We also observe the selective enrichment of POLE3 activity. Being a component of the DNA polymerase subunits, POLE3 is involved in inducing genetic and epigenetic programs in the cells [38]. POLE3 selectively binds to histones H3-H4 together with POLE4, acting as histone chaperones [39]. To our knowledge, no evidence directly suggests the role of POLE3 in cancer epigenetics. Altered expression of *DNMT1* and *POLE3* as observed upon CPT-CEF treatment of the cells could be conjectured as a mechanism to restore the hypomethylation of genes and hence repair faulty switches that lead to aberrant proliferation in HT29 cells.

Global enzyme regulation is dependent on the availability of metabolites as cofactors. In cancers, metabolic rewiring skews the normal profile of metabolites. In turn, these metabolites exert a profound effect on the activity of the cellular enzymes, including those which are involved in chromatin modulation [40]. In colon cancers, the ratio of the metabolites S-adenosylmethionine to S-adenosylhomocysteine (SAH) is also altered, which coordinates the initiation and progression of cancer through epigenetic changes in the DNA and hence modification of gene expression mechanisms. Thus, as an integrated mechanism, metabolic reprogramming in cancers leads to the synthesis of selective metabolites, which may affect the activity of chromatin-regulating enzymes. Hence, both metabolic reprogramming and epigenetic modification work as concerted cause and effect mechanisms towards cancerous progression. Finally, through this study, we conclude that CPT-CEF mediates its anticancer activity through a mechanism that intercepts epigenetic control and metabolic modulation in colon cancer cells. This plausibly skews cancer-induced metabolic deregulation towards metabolic repair. Alterations in the epigenetic program induced through CPT-CEF treatments divert the oncogenic driver towards apoptotic pathways. These epigenetic mechanisms may be harnessed as targets for regenerating normal metabolism in cancers with better treatment potential.

This study has to be interpreted with bioinformatics analyses. Nonetheless, assimilating this knowledge warrants validation of the genes through Real Time-PCR to quantitate the level of expression in the cells. Additionally, it is unclear whether the observed gene expression changes occurred as a response to the treatment or whether they were responsible for inducing cellular apoptosis. Additionally, there is a need to determine the changes in specific gene transcription involved in metabolic reprogramming following deviation from the normal levels of *PKM*, *DNMT* and *POLE3* genes. Further experimental studies including using animal models are necessary to investigate and relate the sequential mechanisms leading to cell death upon exposure to CPT-CEF. Nevertheless, this study has shown the potential of our formulation from a bioinformatics-focused perspective. The magnetic nanocarrier conjugated CPT containing β-cyclodextrin, iron NPs (Fe_3_O_4_) and EDTA cross-links has higher efficacy as an anticancer drug compared to CPT alone. This paves the way for a variety of formulations with similar design concepts.

## 4. Material and Methods

### 4.1. Treatment of HT29 Colon Cancer Cells with CPT-CEF Nanocompound

The CPT-CEF nanocompound stock solution was prepared by dissolving the compounds in 10% dimethyl sulfoxide (DMSO) (Naccalai, Kyoto, Japan) in a complete culture medium. Human colorectal carcinoma cells line, HT29, was procured from the Laboratory of Vaccine and Immunotherapy (LIVES) Institute of Biosciences (IBS), UPM. The cells were seeded at a concentration of 1 × 10^4^ cells/mL of culture medium containing Roswell Park Memorial Institute (RPMI) 1640 Medium (Naccalai, Kyoto, Japan), 10% Fetal Bovine Serum (Naccalai, Kyoto, Japan) and 1% Penicillin/Streptomycin in a 6-well culture plate. The cell culture was maintained at 37 °C in a 5% CO_2_ incubator for 24 h. Following that, the supernatant was removed and CPT-CEF nanocompound solution was added to the culture medium to achieve a final concentration of 133.5 μg/mL (IC_50_). The DMSO content did not exceed 1% of the final concentration of nanocompound in the culture medium. The cell culture was then kept in the CO_2_ incubator for 48 h, after which the RNA was extracted for sequencing. To compare the efficiency of treatment to halt cell proliferation, untreated cells were used as control group. The untreated HT29 colon cancer cells were incubated in the culture medium containing less than 1% of DMSO [20,41]. The experiment was carried out in triplicate for both CPT-CEF treated and untreated cells to ascertain the precision and accuracy of treatment.

### 4.2. Isolation of Total RNA from HT29 Colon Cancer Cells

Total RNA from CPT-CEF-treated and untreated HT29 colon cancer cells (*n* = 3) was extracted using the RNeasy mini kit according to the manufacturer’s protocol (Qiagen, Hilden Germany). The untreated HT29 colon cancer cells were incubated in the culture medium containing less than 1% of DMSO. In brief, 350 µL of RLT buffer supplemented with 5% β-mercaptoethanol was used to lyse the cells. A pipette was used to directly homogenize the samples. An equal volume of 70% ethanol was added to obtain a 1:1 ratio of ethanol and lysate. The mixture was then aliquoted into the RNA spin column and centrifuged at maximum speed (~12,000× *g*) for 15 s. After a brief washing step, the total RNA was eluted using 50 µL of RNase-free water. The purity and RNA integrity were then measured to ensure that all samples had a RIN of at least 7 and an A_260_/A_280_ ratio of approximately 2. The Bioanalyzer 2100 system (Agilent Technologies, Santa Clara, CA, USA) was used to measure RNA integrity, whereas the NanoDrop 2000 Spectrophotometer (Thermo Scientific, Waltham, MA, USA) was used to measure the A_260_/A_280_ ratio.

### 4.3. Library Preparation for RNA-Seq

Library preparation was performed using the NEBNext^®^ Ultra™ II RNA Library Prep Kit for Illumina^®^ (New England Biolabs, Ipswich, MA, USA) according to the manufacturer’s protocol. In brief, mRNA enrichment of total RNA was first performed. In this step, total RNA was incubated with 50 µL of oligo dT beads and binding buffer to bind the poly (A) mRNA strands. The beads were incubated in a thermal cycler at 65 °C for 5 min to denature and facilitate RNA binding. The tubes were then placed onto a magnetic stand to capture the beads. After several washing steps to remove residual rRNA and other RNA species, the beads were mixed with 50 µL of Tris-buffer and incubated in a thermal cycler at 80 °C for 2 min, followed by cooling until 25 °C to elute the mRNA from the beads. The process was repeated to rebind mRNA, but incubation took place at RT (25–30 °C). Final elution was then carried out using the first strand synthesis reaction mix. First-strand cDNA synthesis was the next major step in the protocol, where the isolated mRNA was added to the first strand reaction mix and then incubated for 10 min at 25 °C, 15 min at 42 °C, 15 min at 70 °C, and then put on hold at 4 °C. Second strand synthesis was performed immediately after by incubating the samples with the reaction mix for 1 h at 16 °C. Purification of the double-stranded cDNA was then carried out using NEBNext Sample Purification Beads and magnets to capture the strands. After several washing steps, the beads were left to air dry. The cDNA was then eluted using 53 μL of 0.1× TE Buffer. At the end of this step, 50 µL of the eluent was stored in clean nuclease-free PCR tubes. The next step was end prep, where ligation was performed to attach adaptors to each cDNA library. The ligation reaction mix containing a unique adaptor was mixed with a cDNA library and incubated at 20 °C for 15 min. The ligation reaction was then purified using the NEBNext Sample Purification Beads. Finally, PCR enrichment of adapter-ligated cDNA was performed to expand the library before sequencing. After the enriched libraries were purified using the NEBNext Sample Purification Beads, the quality of the library was assessed using the Bioanalyzer 2100 system. All the samples showed a peak size of approximately 300 bp on the electropherogram with no adaptor primer–dimer peaks and were thus suitable for RNA-seq.

### 4.4. RNA-Seq Data Processing and Annotation

Sequencing was performed using the Illumina HiSeq2000 system (Illumina, Hayward, CA, USA). The read length used in this study was 2 × 100 bp. More than 80% of all the sequenced reads had good quality scores (>Q30) and possessed an average depth of 4 million reads. The FastQC tool was used to assess the quality of the raw reads, and no adaptor sequence contamination was present. The Salmon tool (available at github.com, accessed on 16 January 2021) was then used to map the RNA-seq data to the GRCh38 homo-sapiens reference transcriptome (available at asia.ensembl.org). The raw data of the sequences was deposited in the Gene Expression Omnibus (GEO) dataset (Accession No. GSE165875). Quantification was then performed using Salmon and annotated based on Ensembl IDs.

### 4.5. Differentially Expressed Genes between CPT-CEF-Treated and Untreated HT29 Colon Cancer Cells

Differential expression analysis was performed using the DeSeq2 tool (available at Bioconductor.org). This allowed the quantitated reads to be normalized per sample scaled by the medium of ratio. The raw data comprised 11,118 transcripts. After applying a filter threshold of adj *p* < 0.10 and fold change >2.0, 894 differentially expressed genes (DEGs) were isolated. A volcano plot was used to visualize the differentially expressed genes.

### 4.6. Over-Representation Analysis of Differentially Expressed Genes

Over-representation analysis (ORA) was performed using g:Profiler to profile the DEGs (https://biit.cs.ut.ee/gprofiler/, accessed on 16 January 2021). Homo sapiens RNA sequences were used as the reference. The significance threshold for multiple testing corrections was set at g:SCS, whereas adj *p* < 0.05 was set as the cutoff value. The output file was generated based on data mined from Gene Ontology (GO). The data were categorized based on GO subontologies for molecular function (MF), cellular component (CC), and biological processes (BP).

### 4.7. Functional Enrichment Analysis of CPT-CEF-Treated HT29 Colon Cancer Cells

Functional enrichment was performed using the GSEA tool (https://www.gsea-msigdb.org/gsea/index.jsp, accessed on 16 January 2021) to determine enriched pathways between the treated and untreated samples. The WikiPathways database was used as a reference (c2.cp.wikipathways.v7.2.symbols.gmt). The number of permutations was set to 1000, and the permutation type was set to gene set. Annotation platform was set to Ensembl (Human_ENSEMBL_Gene_ID_MSigDB.v7.2.chip). The metric for gene ranking was set to log_2_ ratio of classes. Apart from these, the rest were set to run at default.

### 4.8. Identification of Associated Genes involved in Epigenetic Modifications

The processed RNA-seq data were used for data mining of associated epigenetics genes from the EpiFactors database. A list was generated based on the HGNC approved name, function, types of epigenetic modification, target molecule, target entity, a product of modification, and finally a brief commentary on the respective epigenetic mechanism. The list was then cross-checked with the GSEA-enriched pathways to identify genes with high correlations to both biological functions.

## 5. Conclusions

In this study, we have identified genes that may play a fundamental role in modulating the transcription of genes involved in metabolic switching. A total of 95 upregulated and 146 downregulated genes were observed. From these, we identified genes that were involved in epigenetic modification in the treated HT29 cells. The top 13 genes were data-mined using EpiFactors and were found to be involved with chromatin remodeling and histidine modification pathways, which highlighted the importance of these mechanisms in CPT-CEF and colon cancer-mediated interaction. A total of 20 top pathways were also uncovered, which showed key cancer pathways such as metabolic reprogramming that are involved in epigenetic modification. Since metabolic reprogramming is a distinct feature of cancer growth, the metabolic genes that are downregulated in CPT-CEF treated cancer cells were likely to stall the progression of these cells toward further proliferation. Indeed, in our previous study, the treatment successfully inhibited in vitro cell growth. Hence, this study could provide avenues that need exploration and affirmation to elucidate the aforementioned mechanisms. The knowledge may lead to the finding of potential targets for reversing colon cancer to normal metabolism in the future.

## Figures and Tables

**Table 1:** A list of the top 10 GO terms obtained from each subontology. Over-representation analysis was performed using g:Profiler (adj *p* < 0.05).

GO Subontologies	GO Term ID	Description	Log_10_ Adj *p*
Molecular function	GO:0003735	Structural constituent of ribosome	−12.0783
	GO:0005198	Structural molecule activity	−3.4232
	GO:0005488	Binding	−3.2484
	GO:0009055	Electron carrier activity	−1.3142
	GO:0050839	Cell adhesion molecule binding	−8.2226
	GO:0019843	rRNA binding	−2.6811
	GO:0043021	Ribonucleoprotein complex binding	−2.0528
	GO:0097159	Organic cyclic compound binding	−8.567
	GO:0044877	Macromolecular complex binding	−4.4486
	GO:0036094	Small-molecule binding	−1.8064
Cellular component	GO:0005576	Extracellular region	−2.9459
	GO:0005740	Mitochondrial envelope	−4.2464
	GO:0005925	Focal adhesion	−7.9031
	GO:0016020	Membrane	−1.5299
	GO:0031974	Membrane-enclosed lumen	−19.5114
	GO:0032991	Macromolecular complex	−13.1798
	GO:0043226	Organelle	−22.2958
	GO:0098589	Membrane region	−2.1022
	GO:1990904	Ribonucleoprotein complex	−14.2526
	GO:0030027	Lamellipodium	−1.9261
Biological function	GO:0006614	SRP-dependent cotranslational protein targeting to Membrane	−16.6126
	GO:0008152	Metabolic process	−9.5346
	GO:0051179	Localization	−7.2899
	GO:0051347	Positive regulation of transferase activity	−1.9564
	GO:0070482	Response to oxygen levels	−2.8992
	GO:0071840	Cellular component organization or biogenesis	−13.6253
	GO:0000184	Nuclear-transcribed mRNA catabolic process, nonsense-Mediated decay	−14.0888
	GO:0016032	Viral process	−11.6289
	GO:0006915	Apoptotic process	−2.367
	GO:0006091	Generation of precursor metabolites and energy	−3.2792

**Table 2:** A list of the top 13 genes from the filtered dataset (adj *p* < 0.05, FC > 2.0) that are involved in epigenetic modifications. The genes were data-mined using EpiFactors as a reference database. FC, fold change.

Symbol	Description	Function	Adj *p*	Log_2_FC
CHD6	Chromodomain helicase DNA Binding protein 6	Chromatin remodeling	2.35 × 10^−2^	0.915414
ARID4B	AT rich interactive domain 4B (RBP1-like)	Histone modification write cofactor	7.71 × 10^−3^	0.912247
KDM5A	Lysine (K)-specific demethylase 5A	Histone modification erase	5.61 × 10^−2^	0.707035
DAXX	Death-domain associated protein	Transcriptional regulation, cell apoptosis, carcinogenesis	2.15 × 10^−2^	−0.13739
CTBP1	C-terminal binding protein 1	Chromatin remodeling	4.62 × 10^−2^	−0.6337
HMGB1	High mobility group box 1	Chromatin remodeling	1.30 × 10^−2^	−0.64422
MTA1	Metastasis associated 1	Chromatin remodeling cofactor	5.82 × 10^−2^	−0.72427
DDX21	DEAD (Asp-Glu-Ala-Asp) box helicase 21	RNA modification	6.45 × 10^−2^	−0.73776
FBL	Fibrillarin	Histone modification write	3.82 × 10^−2^	−0.89229
HDGF	Hepatoma-derived growth factor	Chromatin remodeling, TF	4.99 × 10^−6^	−1.15119
DNMT1	DNA (cytosine-5-)-methyltransferase 1	DNA modification	8.08 × 10^−4^	−1.2368
FBRSL1	Fibrosin like 1	Histone modification	4.16 × 10^−3^	−1.46469
EXOSC5	Exosome component 5	Scaffold protein, RNA modification	1.79 × 10^−3^	−2.01552

**Table 3:** A list of the top 20 enriched pathways in CPT-CEF-treated colon cancer cells. Gene set enrichment analysis was performed using the WikiPathways database as a reference (adj *p* < 0.05). NES, normalized enrichment score.

WikiPathways ID	NES	Adj *p* Value
WP_CYTOPLASMIC_RIBOSOMAL_PROTEINS	−2.53	0
WP_METABOLIC_REPROGRAMMING_IN_COLON_CANCER	−2.28	4.07 × 10^−4^
WP_PYRIMIDINE_METABOLISM	−2.25	2.71 × 10^−4^
WP_GLYCOLYSIS_AND_GLUCONEOGENESIS	−2.19	4.22 × 10^−4^
WP_DNA_REPLICATION	−2.13	1.03 × 10^−3^
WP_BASE_EXCISION_REPAIR	−2.11	1.43 × 10^−3^
WP_DNA_IRDAMAGE_AND_CELLULAR_RESPONSE_VIA_ATR	−2.06	2.09 × 10^−3^
WP_GASTRIC_CANCER_NETWORK_2	−2.00	3.85 × 10^−3^
WP_G1_TO_S_CELL_CYCLE_CONTROL	−2.00	3.42 × 10^−3^
WP_MRNA_PROCESSING	−1.99	3.51 × 10^−3^
WP_ONE_CARBON_METABOLISM	−1.96	4.97 × 10^−3^
WP_DNA_MISMATCH_REPAIR	−1.96	4.63 × 10^−3^
WP_NUCLEAR_RECEPTORS	−1.86	1.76 × 10^−2^
WP_RETINOBLASTOMA_GENE_IN_CANCER	−1.85	1.78 × 10^−2^
WP_TRANSSULFURATION_AND_ONE_CARBON_METABOLISM	−1.79	2.92 × 10^−2^
WP_PURINE_METABOLISM_AND_RELATED_DISORDERS	−1.79	2.93 × 10^−2^
WP_UREA_CYCLE_AND_ASSOCIATED_PATHWAYS	−1.77	3.33 × 10^−2^
WP_PPAR_ALPHA_PATHWAY	−1.74	3.79 × 10^−2^
WP_PHOTODYNAMIC_THERAPYINDUCED_HIF1_SURVIVAL_SIGNALING	−1.74	3.82 × 10^−2^
WP_CELL_CYCLE	−1.69	5.56 × 10^−2^

**Table 4:** A list of GSEA-enriched genes from campthotecin-CEF-treated colon cancer cells in the colon cancer metabolic reprogramming pathway. The genes were obtained and ranked by using GSEA with WikiPathway as the reference map. RMS, Ranked metric score; RES, Running enrichment score.

Symbol	Description	RMS	RES	Core Enrichment
PDHA1	pyruvate dehydrogenase E1 subunit alpha 1	−0.05	−0.61	Yes
ACLY	ATP citrate lyase	−0.05	−0.61	Yes
PPAT	phosphoribosyl pyrophosphate amidotransferase	−0.06	−0.60	Yes
FH	fumarate hydratase	−0.06	−0.59	Yes
MDH2	malate dehydrogenase 2	−0.06	−0.58	Yes
PYCR2	pyrroline-5-carboxylate reductase 2	−0.08	−0.60	Yes
IDH2	isocitrate dehydrogenase (NADP(+)) 2	−0.08	−0.58	Yes
PGK1	phosphoglycerate kinase 1	−0.08	−0.55	Yes
TIGAR	TP53 induced glycolysis regulatory phosphatase	−0.08	−0.53	Yes
SLC2A1	solute carrier family 2 member 1	−0.08	−0.50	Yes
PFKL	phosphofructokinase, liver type	−0.09	−0.48	Yes
GOT2	glutamic-oxaloacetic transaminase 2	−0.09	−0.45	Yes
LDHA	lactate dehydrogenase A	−0.09	−0.43	Yes
PYCR1	pyrroline-5-carboxylate reductase 1	−0.09	−0.40	Yes
GPI	glucose-6-phosphate isomerase	−0.11	−0.38	Yes
PSPH	phosphoserine phosphatase	−0.11	−0.34	Yes
PKM	pyruvate kinase M1/2	−0.11	−0.30	Yes
PGAM1	phosphoglycerate mutase 1	−0.11	−0.26	Yes
SHMT2	serine hydroxymethyltransferase 2	−0.11	−0.22	Yes
RPIA	ribose 5-phosphate isomerase A	−0.11	−0.19	Yes
SLC16A3	solute carrier family 16 member 3	−0.14	−0.15	Yes
GART	phosphoribosylglycinamide formyltransferase, phosphoribosylglycinamide synthetase, phosphoribosylaminoimidazole synthetase	−0.14	−0.11	Yes
ENO1	enolase 1	−0.15	−0.06	Yes
PAICS	phosphoribosylaminoimidazole carboxylase and phosphoribosylaminoimidazolesuccinocarboxamide synthase	−0.19	0.00	Yes

**Table 5:** Several genes involved in colon cancer metabolism were also found to be involved in epigenetic modifications. The genes were data-mined using EpiFactors as a reference database. FC, fold change.

Symbol	Description	Function	Target Molecule	Adj *p* Value	Log_2_FC
PKM	pyruvate kinase, muscle	Histone modification write cofactor	histone	Yes	−1.02522
DNMT1	DNA (cytosine-5-)-methyltransferase 1	DNA modification	DNA	Yes	−1.2368
POLE3	polymerase (DNA directed), epsilon 3, accessory subunit	Histone chaperone	histone	Yes	−0.54009

## Data Availability

Data resources: The datasets presented in this study can be found in the Gene Expression Ominibus (GEO) public repository with accession number GSE165875.

## Abbreviations

CPT-CEF; camptothecin encapsulated in β-cyclodextrin-EDTA-Fe_3_O_4_ nanoparticles, DNMT-1; DNA-methyltransferase 1, POLE3; DNA Polymerase Epsilon 3 Accessory Subunit, PKM-2; Pyruvate Kinase M2, NO; Nitric oxide, NP; Nanoparticles, EDTA; Ethylenediaminetetraacetic acid, FE3O4; Iron(II, III) oxide, GO; Gene ontology, BP; Biological processes, CC; Cellular components, MF; Molecular functions, FC; Fold change, CHD6; Chromodomain Helicase DNA Binding Protein 6, SLC2A1; Solute Carrier Family 2 Member 1 for Glucose Transporter, LDHA; Lactate Dehydrogenase, GPI; Glucose 6 phosphate isomerase, ENO1; enolase 1, PKM; pyruvate kinase, TCA;Tricarboxylic acid pathway, IDH2; Isocitrate Dehydrogenase, MDH2; Malate Dehydrogenase, GOT2; glutamic oxaloacetic transaminase 2, PYCR1 and 2; pyrolline-5-carboxylase reductase, ACLY; ATP citrate lyase, FASN; fatty acid synthase, PAICS; phosphoribosylami-noimidazole carboxylase and phosphoribosyla minoimidazolesuccinocarboxamide synthase.
